# Abstract of Proceedings of the Buffalo Medical Association

**Published:** 1869-09

**Authors:** T. M. Johnson

**Affiliations:** Sec’y.


ART. II.—*Abstract of Proceedings of the Buffalo Medical Association.*

TUESDAY EVENING, SEPT. 1869.

The meeting was called to order by the President.

In response to the call for voluntary communications, DR. JOHNSON related the following case:

A young lady, whose monthly courses had been delayed a few days, mentioned the fact in the presence of a female quack, who advised her to procure ten cents worth each of the dried leaves of foxglove and tanzy, steep them together in about a quart of water, and drink the whole decoction in the evening before going to bed. The patient did as directed, and procured an ounce package of each of the above articles, steeped them in less than a quart of water, and drank half a pint of the decoction at a single draught. She soon began to feel sickness at the stomach, complained of headache and faintness. A gentleman of the family, who had considerable knowledge of medicine, on learning the facts of the case, gave her largely of warm mustard water, which produced quite copious emesis about four hours after the drug had been taken. I saw the patient about this time and found her vomiting almost constantly, complaining of great distress at the stomach, and nausea pain in the head, and confusion of ideas, scarcely knowing what was going on around her. Pulse about fifty and very irregular. The gentleman mentioned above told me, at this time, that the pulse was less than forty per minute an hour before I saw her, prostration very great. As nothing would be retained in the stomach for more than two or three minutes, morphine, by hypodermic injection seemed to me to be the most proper treatment, and I first injected half a grain, and an hour and a half later a fourth of a grain. This brought on quietude and considerable sleep during the night and a cessation of the vomiting. The next morning, however, the vomiting returned. The pulse was, at this time, a hundred and twenty and irregular—ordered iced champagne, a tablespoonful every half hour. This allayed the quite intense thirst and seemed to control the vomiting. The next day the pulse was much stronger, more regular, and the patient much improved, and now, on the fifth day, is probably out of danger and has had a very narrow escape from death. She must have taken fully two drachms of the dried and well cured leaves of digitalis. Her courses came on twenty-four hours after taking the medicine.

Dr. E. R. Barnes said that a case had recently come under his observation where the cumulative effect of digitalis was developed from taking nine doses of infusion of the leaves at intervals of two hours. The infusion contained two scruples of the leaves in a pint of water. The dose taken was a desert-spoonful. After the seventh dose copious diuresis was produced. After the ninth dose the patient was suddenly overcome by a sense of extreme prostration with coolness of the extremities and profuse cold prespiration. The pulse was irregular and weak, though at first perhaps increased in volume. It was reduced to forty beats per minute. Stimulants were used, and, after some hours, improvement was apparent in the general condition, but the pulse remained at forty, with much physical and mental depression, for eight days.

Dr. Miner presented a specimen of exfoliation of the femur, after amputation of the thigh, at the junction of the lower with the middle third. The end of the bone, where cut by the saw, extending upwards about half an inch and then gradually thinning towards the medullary canal, coming to a point about five inches above the amputation, had exfoliated and been extracted entire from the stump. After this dead bone was removed the wound healed rapidly, and in a few weeks the stump was in all respects perfect. New bone had been deposited, the end had rounded off smoothly, and the result of amputation satisfactory. The specimen was presented, not so much on account of rarity, for such accidents are common, but on account of its size, and as showing very beautifully one of the accidents liable to follow amputation. It is not a very serious accident, but delays recovery and exposes to greater danger of purulent absorption. The one question concerning it of greatest interest is, what are the causes of such death of bone after amputation? You will observe that the whole bone, in this instance, looses its vitality back from the point of division full half an inch, and that from this point it extends towards the medullary canal. Without proposing any very extensive discussion of the causes of this death of bone, it may be well to remark, that it appears as though the periosteum had been separated from the bone and that death resulted to the extent of this separation. Bone thus robbed of its periosteal covering does not always die, but it certainly lessens its vascular supply and appears to have an important influence in causing its death. Disturbance of the medullary canal may also diminish vascular supply and contribute to the same end. The appearance of the exfoliation, its shape and extent, are all suggestive of a practical point of some value.

It may be, and probably is true, that surgeons take too little care in dividing the periosteum and bone. The procedure is conducted with too much haste. The membrane surrounding the bone is often divided with the saw and thus torn, and often separated from the bone to some extent above the point of division. This is not always followed by necrosis of bone; but this, joined to a depressed and very low condition of the system is often followed by necrosis. All the specimens thus far obtained, of much magnitude, were obtained from patients who, for weeks after operation, suffered from dangerous and protracted depression of the vital forces. The patient from whom this specimen was obtained presented this condition in a marked degree.

Another specimen, very singular in appearance and of equal length, was exhibited, which had been recently obtained from a patient upon whom, three years before, he had made exsection of the elbow joint for injury. The patient was unwilling to suffer amputation, choosing rather to accept the vastly greater risks of exsection. The injury to soft parts and bone was fearful, but the radial pulse could be discovered, and he reluctantly consented to make exsection, with little expectation, however, of saving the forearm. The patient passed from his care to the Sisters' Hospital, and was carefully watched by the attending surgeons. The patient regained considerable use of the arm, and, after protracted suppuration and exhaustion, also gradually regained nearly his former health and vigor. In about three years he again came under care for relief, now willing to suffer amputation, if thought necessary, in order to obtain freedom from pain and suppuration. Upon examination this dead bone was found within an extensive deposit of new bone. By cutting out with trephine a part of this new deposit, the dead bone was removed entire. Pain and suppuration soon ceased, the wound healed very kindly, and the result is satisfactory. The arm is now a remarkable relict of conservative surgery, possessing motion and strength in very useful degrees.

An informal discussion then followed, in which the members present participated, upon the various questions involved, such as the causes, frequency, effects and modes of preventing such accidents after amputations or other operations upon bone.

On report of prevailing diseases, Dr. Barnes stated that the cases of cholera-morbus he had seen the present season were of a severe type, being attended by cramps in the extremities, and followed by marked prostration. Had seen one patient die in the congestive or typhoid state. He asked whether any of the members had observed that this congestive stage followed one method of treatment rather than another, either in cholera-morbus or cholera.

None of the members present expressed themselves as having discovered any difference in this respect.

Dr. Johnson remarked that he had seen several cases of cholera-morbus of unusual severity, some of which were little short of epidemic cholera.

Dr. Taylor said that he saw in the Island hospital three hundred cases of cholera among six hundred occupants in the workhouse. Two hundred of them died—many of them did not have true cholera. Death occurred in most of the cases in the congestive stage. The results of treatment by hypodermic injection were more unfavorable than by other modes of treatment.

Dr. E. R. Barnes was elected to read an essay at the next meeting.

Adjourned. T. M. Johnson, *Sec'y.*

